# Piezoelectric Bone Conduction Hearing Implant: A Case Series of Audiological, Surgical and Patient-Reported Outcomes

**DOI:** 10.3390/jcm13113111

**Published:** 2024-05-26

**Authors:** Mai Vagle, Michael Bille, Ramon Gordon Jensen

**Affiliations:** 1 Faculty of Health and Medical Sciences, University of Copenhagen, 2200 Copenhagen, Denmark; 2 Copenhagen Hearing and Balance Center, Department of Otorhinolaryngology, Head and Neck Surgery & Audiology, Copenhagen University Hospital, 2100 Copenhagen, Denmark

**Keywords:** bone conduction, hearing loss, osseointegrated steady-state implant (OSIA), mixed hearing loss, conductive hearing loss, single-sided deafness

## Abstract

**Objective**: To examine the surgical, audiological and patient-reported outcomes of the Osia 2 implant. **Methods**: Data from 14 consecutive subjects undergoing implantation between April 2022 and November 2023 were reviewed. Ten subjects had conductive hearing loss, three had mixed hearing loss and one had single-sided deafness (SSD). Warble tone thresholds, Pure Tone Average (PTA_4_) and Speech Discrimination Score (SDS) in quiet and in noise were determined unaided and aided. The subjective outcome was determined from two standardized questionnaires: (1) International Outcome Inventory for Hearing Aids (IOI-HA) and (2) Speech, Spatial and Qualities of Hearing Scale 12 (SSQ12b). **Results**: Unexpected postoperative pain was found in four cases. The warble tone thresholds exhibited a consistent reduction across all frequencies, contributing to a mean decrease of 27 dB in the aided PTA_4_. SDS demonstrated notable improvements, with a 57.3% increase at 50 dB and a 55.6% increase at 65 dB. In noise, SDS exhibited a 43.9% improvement. The mean IOI-HA Score was 3.8, and the mean overall score for SSQ12b was 6.6, with consistent findings across the subgroups. **Conclusions**: The Osia device emerges as a promising recommendation for individuals with conductive or mixed hearing loss, possibly also for those with SSD. Its safety and efficacy profile aligns with the broader category of active transcutaneous devices, demonstrating a reduced risk of wound infection compared to percutaneous alternatives. Both audiological assessments and subjective evaluations revealed positive outcomes.

## 1. Introduction

The significance of bone conduction hearing implants (BCHIs) has grown considerably in recent years, particularly for patients with conductive or mixed hearing loss, as well as those with single-sided deafness (SSD) [1]. BCHIs are currently categorized into passive percutaneous, passive transcutaneous and active transcutaneous devices [2,3]. For a significant period, passive bone conducting systems, which comprise an external sound processor and a transducer positioned behind the ear, have dominated the field. In these systems, the transducer-induced vibration is transmitted to the implanted component inside the bone either through a percutaneous abutment in percutaneous devices or through a magnet system in transcutaneous devices. However, both types of passive devices have their respective drawbacks. The passive percutaneous devices break the skin barrier, increasing the risk of local skin complications such as infections and irritations, necessitating lifelong daily care. Conversely, passive transcutaneous devices use a magnetic attraction force and do not cause permanent skin defects. Nevertheless, the quality of the sound can be limited because of the sound attenuation caused by the skin between the magnets, and the continuous pressure on the skin may lead to pain and redness [4,5].

Over the past two decades, several active bone conducting systems have emerged on the market, offering a possibility to avoid skin discontinuity and soft tissue attenuation [2,6,7]. These systems feature a transducer located within the patient’s bone, with the processor attached using magnetic attraction forces. The first electromagnetic device, Bonebridge (Med-El, Innsbruck, Austria) was introduced in September 2012 [8]. In 2019, the Osia (Cochlear, Sydney, Australia) obtained European Conformity (CE) approval as the first active bone conducting system with a piezoelectric transducer. The principle of piezoelectricity involves the transducer undergoing deformation and generating vibrations when exposed to electric signals from the external sound processor. These vibrations are subsequently transmitted through the bone tissue to the cochlea. In October 2021, Cochlear launched a new Osia 2 system, which is smaller in size and incorporates additional features such as automated setting on microphones, wireless compatibility, adjustment of settings through a mobile app and extended battery life [6,9]. Bonebridge has been offered to patients at the Copenhagen Hearing and Balance Centre since 2013 [10], and Osia^®^ 2 since 2022. 

The objective of this study was to examine the surgical efficacy, the hearing performance and patient-reported outcomes of the Osia 2 implant in the first 14 patients in Denmark. This investigation aimed to enhance our understanding of the usefulness of the new implant for patients with conductive or mixed conductive and sensorineural hearing loss, as well as those with SSD. 

## 2. Materials and Methods

### 2.1. Study Design and Outcome Measurements

The study was conducted as an observational, non-interventional case series of 14 patients. The surgical outcome was observed from the medical records by registering the number of surgical complications and device malfunctions. All the patients were examined 10 days after implantation for a wound revision and after 3 months to control for possible postoperative complications. 

For the audiological outcome, sound field hearing was evaluated in a soundproof booth, both unaided and aided, to calculate the functional gain. The thresholds for warble tones at specific frequencies (250 Hz, 500 Hz, 1000 Hz, 1500 Hz, 2000 Hz, 3000 Hz, 4000 Hz, 6000 Hz and 8000 Hz) were ascertained. Unlike pure tones, which maintain a constant frequency, warble tones fluctuate around a central frequency with the purpose of preventing standing waves and the risk of acoustic feedback in the environment. A pure tone average (PTA_4_) was calculated as mean values for the frequencies: 500 Hz, 1000 Hz, 2000 Hz and 4000 Hz. Additionally, the Speech Discrimination Score (SDS) in free field in quiet was determined at 50 and 65 dB sound pressure levels (SPLs) unaided and aided with phonetically balanced monosyllable word lists (Dantale) using the phoneme scoring method [11]. The monosyllable word lists were also used for the SDS in noise at a noise level of 65 dB SPL (signal to noise ratio SNR = 0 dB) unaided and aided. To mimic a daily life situation as close as possible, speech was presented from a front speaker (0 degrees azimuth), with noncorrelated speech-shaped noise (65 dB SPL) presented from speakers placed at ±45 degrees azimuth. The SDS test was selected because it provides a quantitative measure of an individual’s ability to discriminate between different speech sounds. This ability is crucial for understanding speech in everyday listening environments, making SDS a relevant measure of hearing aid performance in real-world conditions.

Among the subjects with hearing in both ears, the non-test ear was masked. The masking was performed by presenting noise through an insert earphone placed on the non-test ear. The masking was performed with 45 dB and 60 dB Hearing Levels (HLs) for the SDS in free field at 50 dB SPL and 65 dB, respectively. For the patient with SSD, the non-test ear was masked with a plug and earmuff. For the one patient with bilateral conductive hearing loss (patient 2) masking was not possible. Patients wearing a hearing aid on the non-test ear had it removed. 

The patient-reported outcome measures were conducted through the utilization of two standardized questionnaires: the International Outcome Inventory for Hearing Aids (IOI-HA) and the Speech, Spatial, and Qualities of Hearing Scale 12b (SSQ12b). The IOI-HA questionnaire consists of 7 questions with the purpose of measuring the effectiveness of hearing aid treatment on a scale from 1 to 5 [12,13]. The SSQ12b consists of 12 questions focusing on everyday scenarios, assessing the patient’s capacity to perceive speech, localize sound sources and appraise the quality of auditory inputs [13]. In the 12 questions, the participants were asked to rate their hearing ability when using the Osia device on a scale from 0 to 10 in comparison to pre-implantation. A score of 10 signified a substantial increase in hearing ability, 5 indicated no change and 0 reflected a significantly diminished hearing ability compared to pre-surgery. 

The follow-up duration for the audiometric and the subjective assessments varied between 1 and 18 months after implantation, with a mean follow-up period of 10.8 months.

### 2.2. Subjects

The study included patients who underwent surgery between April 2022 and November 2023 in Copenhagen. There was a deliberate absence of exclusion criteria to prevent any potential selection bias. However, one patient with autism spectrum disorder was excluded due to challenges in participating in the self-reporting questionnaires. 

Among the 14 consecutive patients who underwent implantation, ten were female, and four were male. Ten exhibited a pure conductive hearing loss, three had a mixed hearing loss, and one had SSD. Three of the patients had bilateral mixed hearing loss (patients 1, 8 and 10), one of the subjects had bilateral conductive hearing loss (patient 2), while the other ten patients had unilateral hearing loss. The mean age at the time of surgery was 41.7 years, with an age range spanning from 17 to 65 years (Table 1).

### 2.3. Surgical Technique and Processor Fitting

All surgeries were performed under general anesthesia. The implant utilizes a piezoelectric actuator anchored to the mastoid bone through an osseointegrated screw. First, the planned incision and the planned position of the implant and the titanium screw were marked. Initially, a retroauricular hockey incision was employed in six cases (patients 1–5 and 8) as recommended by the manufacturer. However, this surgical access was modified during the study, and a retroauricular reverse U incision was utilized in five cases (patients 6, 9, 10, 11 and 13), a posterior C incision was employed in two cases (patients 7 and 14), and an S incision was performed in a singular case among the patients (patient 12). The titanium screw was inserted using a bonebed indicator, and the piezoelectric actuator was positioned on top of the screw.

The patients met for the first processor fitting 5–7 weeks after implantation. Here, the force of the magnet was chosen based on the curve of the skull and the thickness of the skin. For the calibration of the implant, the programming hardware interface, HI-PRO Box, was used. The second processor fitting was performed five weeks after the first fitting. Based on the feedback from the patient, the settings of the processor were optimized.

### 2.4. Statistics

Statistical analyses were conducted using Rstudio Desktop version 2023.09.1 (Posit, PBC, Boston, MA, USA). Statistical significance was set at a *p*-value threshold of ≤0.05. Given that this study consisted of a series of case studies, each subject’s data were analyzed individually to account for the unique characteristics and variability within each case. The non-parametric paired Wilcoxon test was employed to compare the aided and unaided conditions for the warble tone thresholds, pure-tone average at four frequencies (PTA_4_) and speech test results. This method allows for the assessment of within-subject differences and provides a robust analysis of the effectiveness of hearing aids across different audiometric measures.

## 3. Results

### 3.1. Peri- and Postoperative Complications

During the surgical procedures, no major complications occurred. Incisional tension at the closure of the skin was noted at the first surgical procedures, especially in patients with scar tissue from previous ear surgery. Hence, the incision was changed from a direct skin incision to the periost, to a staggered musculoperiostal layer approximately 1 cm from the skin incision. The musculoperiostal flaps were closed separately over the implant to relieve the tension from the skin layer. All the patients underwent a postoperative assessment at two specific intervals, with a 10-day evaluation to assess the condition of the surgical site and a follow-up at 3 months post-surgery to detect any potential postoperative complications. 

In terms of postoperative patient outcomes, four individuals reported unexpected and elevated levels of postoperative pain. All had undergone previous ear surgery and had high tension on the skin. The pain lasted for 14 to 16 days and did not respond adequately to standard pain management with paracetamol and ibuprofen and was supplemented with tramadol. 

One patient experienced bleeding from the scar within the first 48 h post-surgery. Subsequently, an additional examination was conducted on the second postoperative day, during which prophylactic antibiotics were administered. Upon the 10-day assessment of the wound, no indications of infection or compromised wound healing were observed. 

Regarding device-related issues, one patient (patient 12) encountered sporadic attenuation of high-frequency auditory stimuli three months post-surgery. Despite the replacement of the processor on two occasions and recurrent recalibrations, the persisting issue remains unresolved. 

In addition, one patient (patient 14) reported a performance decrement which could not be verified by the technician. Initially, the device operated within expected parameters, but after nine months, it exhibited issues characterized by persistent low sound output despite maximal amplification settings. Despite the concerted efforts involving multiple calibrations and setting adjustments by both technicians from the department as well as the manufacturer, a satisfactory resolution could not be achieved. Due to the low benefit of the device, there was an agreement with the patient to explant and re-implant a new device. Follow-up examinations of the explanted device did not reveal any malfunctions. The patient has been fitted with a new device, but still experiences low benefit. Again, a new series of technical examinations of the new device has been performed. Furthermore, the audiological assessment of the patient’s hearing in the test setting showed improved auditory outcome aided compared to unaided.

### 3.2. Audiological Results

**Figure 1:**
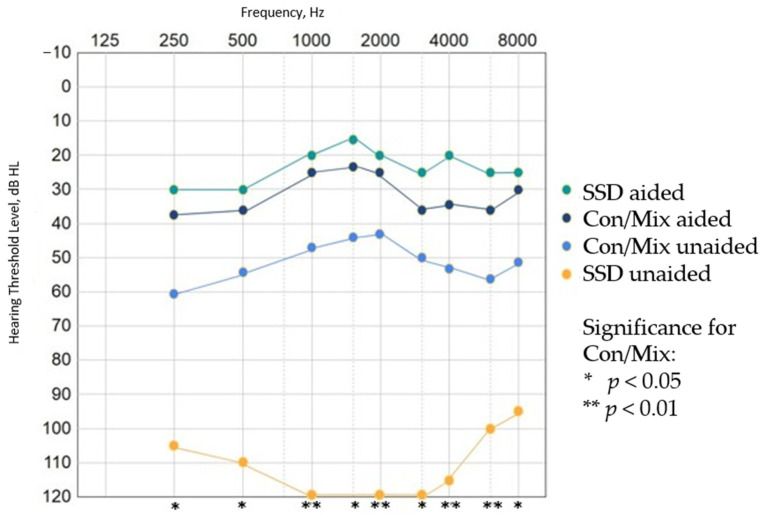
Mean warble tone sound field thresholds for the 13 patients with conductive/mixed hearing loss (Con/Mix) and for the single patient with single-sided deafness (SSD) post-implantation.

The mean warble tone thresholds within the sound field exhibited a reduction when utilizing the Osia device in comparison to the unaided condition across all frequencies, for both the patients with conductive/mixed hearing loss (*n* = 13) and the SSD patient (*n* = 1) (see Figure 1). Given the small sample size and the nature of case studies, the results for the Con/Mix group are presented as an aggregate with individual variability accounted for, while the SSD patient is presented separately due to the uniqueness of their condition. The mean overall improvement in PTA_4_ was 27.0 dB (SD ± 19.1) in the aided group compared to the unaided group, going from 56.8 dB (SD ± 22.9) to 29.7 dB (SD ± 11.8) (*p* < 0.01). 

### 3.3. Verification and Variability

**Figure 2:**
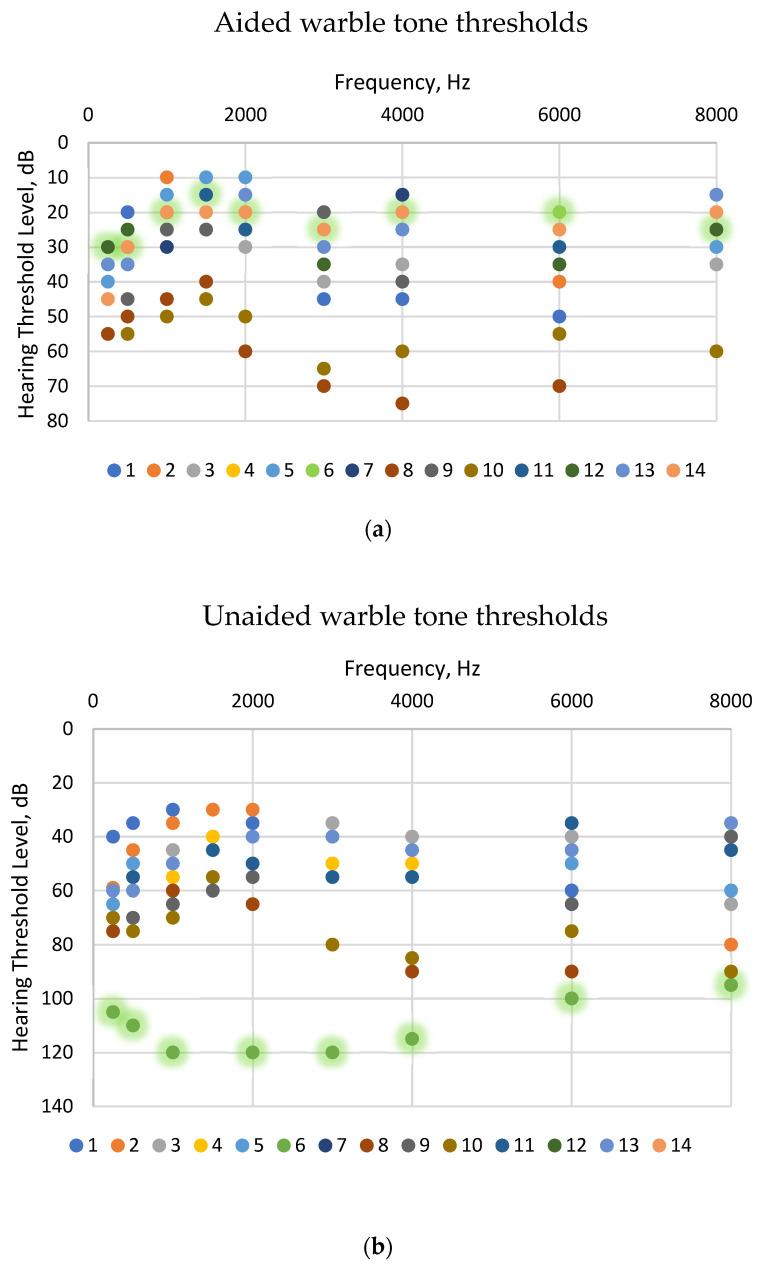
Warble tone threshold for the 14 patients post-implantation. Patient 6, with single-sided deafness is marked with green contour. Patients 1, 8 and 10 had a mixed conductive and sensorineural hearing loss, and the rest had conductive hearing loss. (**a**) Aided warble tone thresholds for the 14 patients.; (**b**) Unaided warble tone thresholds for the 14 patients.

The rationale for grouping the conductive and mixed hearing loss patients together was based on their similar audiological profiles, which allows for a more robust statistical analysis despite the inherent variability in individual cases. This grouping helps to generalize the findings while acknowledging the variability within the group. The detailed results for individual patients in the Con/Mix group are provided in Figure 2 to illustrate this variability and enhance the interpretation of the aggregate data. The single SSD patient was analyzed separately due to their distinct audiological characteristics, which were not comparable to those of the Con/Mix group.

### 3.4. Speech Discrimination Score

**Figure 3:**
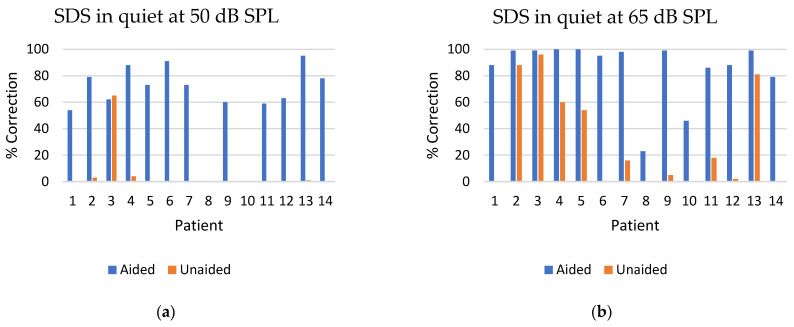
(**a**) Speech Discrimination Score (SDS) for the 14 patients in quiet at 50 dB SPL aided and unaided post-implantation. (**b**) Speech Discrimination Score (SDS) for the 14 patients in quiet at 65 dB SPL aided and unaided post-implantation.

The Osia device demonstrated a statistically significant improvement in SDS in quiet at both 50 dB SPL and 65 dB SPL when compared to the unaided condition (*p* < 0.01). The mean enhancement in SDS in quiet at 50 dB SPL within the aided group, as opposed to the unaided group, was observed to be 57.3% (SD ± 40.5) transitioning from 5.2% (SD ± 17.3) unaided to 62.5% (SD ± 29.3) aided. Similarly, at 65 dB SPL, the mean increase in SDS in quiet in the aided group, in comparison to the unaided group, was 55.6% (SD ± 39.3) ascending from 30.0% (SD ± 37.3) unaided to 85.6% (SD ± 23.0) aided (Figure 3).

**Figure 4:**
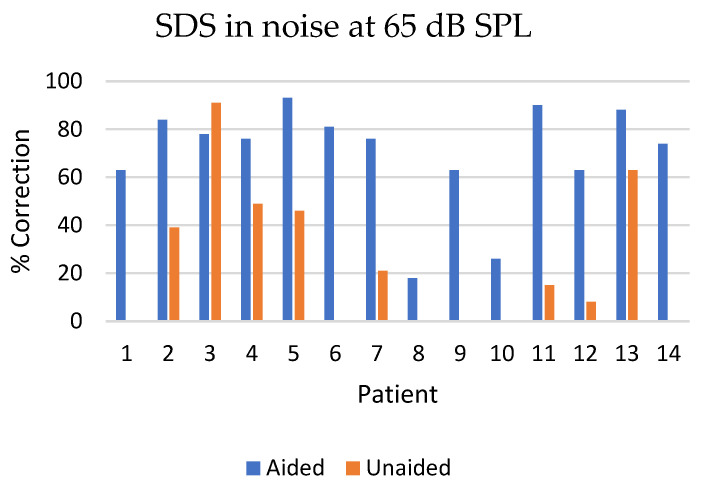
Speech Discrimination Score (SDS) for the 14 patients in noise at 65 dB aided and unaided post-implantation.

SDS at 65 dB SPL in noise showed statistically significant improvement when aided compared to unaided (*p* < 0.01). The mean increase was 43.9% (SD ± 31.1) going from 25.5% (SD ± 29.6) unaided to 69.5% (SD ± 22.3) aided (Figure 4). 

### 3.5. Questionnaire Results

In the study cohort, device usage patterns varied, with 29% (*n* = 4) utilizing the device for more than 8 h daily, 43% (*n* = 6) using it 4–8 h daily, 21% (*n* = 3) using it 1–4 h daily and 7% (*n* = 1) not using the device at all allegedly due to a lost battery, where the patient had not contacted the clinic to have it replaced.

**Figure 5:**
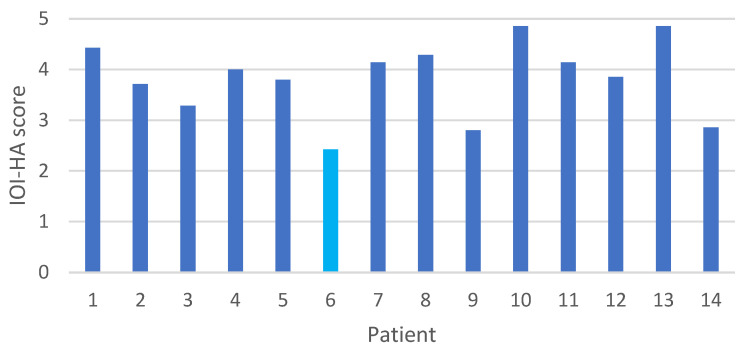
The IOI-HA score for each patient post-implantation. Patient 6, marked in light blue, had single-sided deafness.

Subjective outcomes, as assessed through the IOI-HA questionnaire on a scale from 1 to 5, revealed a mean score of 3.8 (SD ± 0.7). In the Con/Mix group the mean score was 3.9 (SD ± 0.7) and for the SSD patient the mean score was 2.4 (Figure 5).

**Figure 6:**
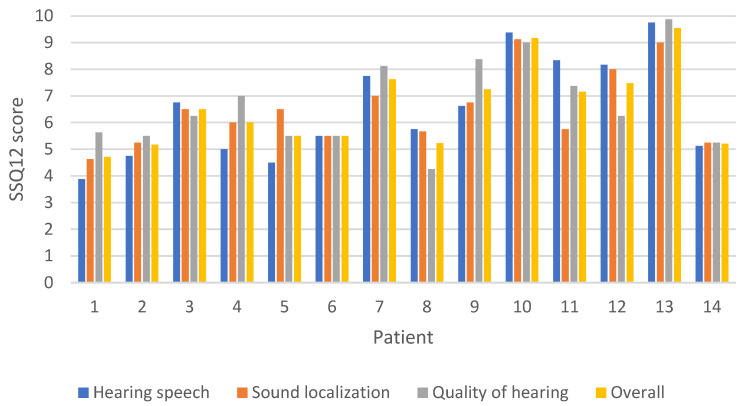
The Speech, Spatial and Qualities (SSQ) scores for each patient, including the overall score and the three subcategories of hearing speech, sound localization and quality of hearing. The questionnaire follow-up was conducted post-implantation, with a mean follow-up time of 10.8 months after surgery.

The overall SSQ12b score measuring the patients’ ability to hear speech, localize sound and identify the quality of sound input on a scale from 1 to 10 was 6.6 (SD ± 1.5). The patient with SSD exhibited an overall SSQ12b score of 5.5 compared to 6.6 (SD ± 1.5) in the Con/Mix hearing loss group. In the Con/Mix group the “hearing speech” component of the SSQ12b was scored as 6.6 (SD ± 1.9), the sound localization was scored as 6.6 (SD ± 1.4) and the “quality of hearing” was scored as 6.8 (SD ± 1.6). For the SSD patient the mean value was 5.5 in all three domains (Figure 6).

## 4. Discussion

This case series of the first 14 patients who underwent implantation with the new active transcutaneous hearing device, Osia 2, in Denmark showed improvement in surgical, audiological and patient-reported outcome measures.

### 4.1. Surgical Efficacy

The surgical outcome revealed no major complications, aligning with the results from the recent published systematic review by Key et al. [3], comprising 14 studies on Osia devices, which indicated a risk profile similar to that of other transcutaneous devices, with a low infection and explantation rate.

However, in this study, unexpected postoperative pain was observed in four cases. Notably, all had undergone previous ear surgeries, a potential explanatory factor, as fibrosis reduces the elasticity of the tissue and therefore poses a recognized risk of skin tension. The unexpected pain prompted increased perioperative vigilance and enhanced patient education regarding the potential for increased postoperative pain. Our center has not encountered similar issues with other devices like BAHA Attract (Cochlear, Sydney, Australia), Bonebridge and Cochlear Implants. To our knowledge, there are no published articles specifically addressing postoperative pain in Osia procedure recipients. The closest relevant study by Lassaletta [14] explored the postoperative pain in patients with active transcutaneous Bonebridge implants, revealing no significant increase in postoperative pain compared to the middle ear and cochlear implant procedures. The study by Szabo et al. [15] compared the percutaneous BAHA Attract to Osia regarding the prolonged pain and numbness during processor use, where significantly lower numbness was observed around a processor with Osia compared to BAHA Attract.

In our study, 5 out of 14 cases reported surgical complications (4 cases of postoperative pain and 1 case of bleeding from the scar). Additionally, there was 1 case of device malfunction, possibly due to a defective processor. Furthermore, there was a singular occurrence of a low sound output where the exact etiology remains undetermined. This finding is noteworthy when compared to prior research. Crowder et al.’s study [16], based on 83 self-reports to the FDA MAUDE database, investigated adverse events post-implantation of either a Bonebridge or Osia device. Of the reported adverse events (91 in total), 34 were associated with Osia devices. Interestingly, Osia had fewer self-reported device malfunctions and more patient injury reports, contrasting with the Bonebridge group. A crucial consideration when interpreting these results alongside Crowder et al.’s study is the inherent limitation of self-reporting systems, as evident in the FDA database, introducing the challenge of potential underreporting.

In light of our findings, it is discerned that the Osia system is emerging as a secure substitute to percutaneous BCHI, a corroboration consistent with antecedent investigations [1,9,17,18,19,20]. Despite the apparent infrequency of severe complications, it is crucial to emphasize the need for extended periods of observation and an enlarged study cohort to augment the statistical robustness of our findings.

### 4.2. Audiological Benefit

The audiological assessments revealed enhanced auditory outcomes in patients with conductive and mixed hearing loss, as well as the patient with SSD. The warble tone thresholds were lowered at all frequencies in the aided situation compared to unaided. When interpreting the results for the mean warble tone threshold from patients with conductive/mixed hearing loss and the patient with SSD in Figure 1, it is essential to consider the variability illustrated in Figure 2. Figure 2a shows that in the Con/Mix group, two patients (patients 8 and 10) had higher aided warble tone thresholds compared to the rest of the group. Both patients had mixed conductive and sensorineural hearing loss. In the unaided situation illustrated in Figure 2b, patient 8 had a higher unaided warble tone threshold at the frequencies of 2000–6000 Hz. In addition, Figure 2b illustrates that the patient with SSD (patient 6) had a higher warble tone threshold in the unaided situation compared to the rest of the patients. 

When examining the speech discrimination score (SDS) in quiet at 50 dB and 65 dB SPLs, the ability to correctly reproduce words from the speaker was higher in the aided situation compared to the unaided situation. At the lower sound intensity (50 dB SPL), patients 2, 3, 4 and 13 were able to reproduce some words correctly in the unaided condition, whereas the remaining patients had a 0% correct reproduction rate when unaided. Notably, patient 3 exhibited better performance in the unaided situation compared to the aided condition at a 50 dB SPL, an observation that remains unexplained. Patient 3 also exhibited a higher SDS at 65dB in noise when unaided compared to when aided. Patients 8 and 10 achieved 0% correct word reproduction in both the unaided and aided conditions. Both patients presented with bilateral hearing loss (see Table 1) and had higher warble tone thresholds than the other patients (Figure 2a), which may explain their poor performance. As anticipated, the SDS was higher at a 65 dB SPL in both the aided and unaided conditions. Nonetheless, there was a significant improvement in SDS when aided compared to unaided. 

In contrast to previous investigations concerning the Osia, our study exhibited a diminished representation of patients with SSD, constituting only 1 out of 14 patients. This disparity arises from prior experiences within the department, wherein BCHIs demonstrated suboptimal efficacy for individuals with SSD. 

The mean pure tone threshold improved by 27 dB from unaided to aided, which is in the same range as previous studies. In the study of 20 recipients of the Osia 2 by Cowan et al. [17], the PTA_4_ improved by 26.7 dB when comparing pre-implantation to 12 months post-implantation. In the study by Briggs et al. [18] of 27 Osia recipients, the PTA_4_ improvement was 28.4 dB from unaided to 6 months aided.

In contrast to percutaneous BCHI, the new active transcutaneous BCHI is anticipated to yield heightened auditory outcomes, particularly at higher frequencies, as elucidated by our findings. As illustrated in Figure 1, a sustained enhancement is discernible in mean warble tones, particularly within the higher frequencies exceeding 2000 Hz. This is consistent with previous research. From the study by Goldstein et al. [21], which compared 6 patients implanted with Osia with 17 patients implanted with BAHA Attract, the additional gain in the Osia patients at 6000 Hz was 19.3 dB compared to BAHA Attract. Kim et al. [22] found a statistically significantly larger audiological gain for Osia, especially at higher frequencies, compared to BAHA Attract and Bonebridge (Med-El, Innsbruck, Austria).

A recognized functionality of the Osia system is the capacity to attenuate non-speech-related noise, which could impact the warble tones. Due to the potential reduction in the perceived loudness of warble tones facilitated by the Osia implant, the thresholds for aided warble tones may have been established at higher sound intensities than intended. For future warble tone threshold establishments, the noise reduction should be turned off in order to prevent the potential reduction in the loudness of warble tones.

In the realm of free field audiometry, the effective masking of the non-test ear poses a challenge. In this investigation, we addressed this challenge by employing an insert earphone during the masking process to obtain the most accurate estimate of the hearing benefit associated with the Osia device, thereby minimizing the potential confounding impact of the contralateral ear. Nevertheless, uncertainty persists regarding the degree to which the non-test ear may have contributed to the observed test results in some cases. Patient 2 demonstrated a bilateral conductive hearing loss, making the traditional masking procedure impossible due to the masking dilemma. 

### 4.3. Patient-Reported Outcome Measures

Given that the duration of device usage serves as a reasonable measure of subjective outcomes, this was probed in the initial question of the IOI-HA questionnaire. The responses revealed that 43% of the patients utilized the Osia device for 4–8 h daily, while 29% reported usage exceeding eight hours per day. It is noteworthy that patient 6, diagnosed with SSD, indicated using the device for 1–4 h daily, possibly influenced by not achieving binaural hearing. In comparison to other studies, Rauch et al.’s study [9] involving 22 subjects (3 with SSD, 19 with conductive hearing loss) reported that all the participants self-reported device usage exceeding 8 h per day during a 12-month follow-up. Briggs et al.’s investigation [18] involving 27 subjects reported an average patient-reported daily usage of the Osia at 8.6 h (ranging from 0.0 to 17.0 h/day) at the 6-month follow-up. It is essential to exercise caution in making direct comparisons due to the potential bias arising from different population sizes and follow-up durations. 

From the IOI-HA questionnaire, the mean score was 3.8, which is considered beneficial according to Cox and Alexander’s study [12]. In their study of subjects with conventional hearing aids, scores of 1 or 2, or less than 20 in total (average score ≤ 2.9), were indicative of pessimistic outcomes. In their study, less than 15% of the individuals reported scores this low. A total score of 33 or more (average score ≥ 4.7) indicated a score in the top 10% of outcomes. Looking at the IOI-HA score from our study, only three patients (patients 6, 9 and 14) had a mean score ≤ 2.9. Patient 9 was a nonuser due to a lost battery, which explains the low IOI-HA score (Figure 3). The fact that this patient did not collect replacement batteries is surprising and underlines the importance of postoperative follow-up and support for some patients after surgery. Patient 6 was the only SSD patient in this study, and although audiological results showed benefit, the patient had the lowest score on the IOI-HA questionnaire of all the 14 patients (Figure 3). A lower IOI-HA score in SSD patients compared to patients with conductive or mixed hearing loss correlates with what was observed in the study conducted by Eberhardt et al. [10]. Patient 14 was the patient being reimplanted due to low sound from the processor, which may have affected the subjective outcome of the hearing device. Although three of the patients had a nonbeneficial mean score, we concluded that the majority of the subjects experienced a benefit from their Osia device. Two subjects (patients 10 and 13) had an average score of ≥ 4.7. 

The overall SSQ score demonstrated an improvement in speech, spatial and sound quality post-implantation. The observed lower score in the three domains for the patient with SSD compared to the patients with conductive or mixed conductive and sensorineural hearing loss, aligns with the findings in the study by Rauch et al. [9]. Furthermore, there was no discernible variation in benefits among the SSQ subgroups compared to the overall SSQ score, consistent with the findings reported by Rauch et al. [9]. However, caution is advised when comparing these studies, particularly given that our study included only one patient with SSD.

While IOI-HA and SSQ12b provide valuable insights into the effectiveness of the Osia device from the patients’ perspective, it is essential to acknowledge that these questionnaires rely on patients recalling their hearing abilities before surgery. This introduces the potential for recall bias, a recognized limitation associated with questionnaire-based assessments.

### 4.4. Strengths and Limitations 

A notable strength of this study lies in the systematic collection of interdisciplinary data from the outset. Additionally, the 3-month post-surgery follow-up served as an ongoing evaluation point, allowing for continuous assessment and modification of the treatment approach. All the patients were able to report any concerns or issues not covered by the questionnaires during this follow-up. The primary limitation of this study is the relatively short follow-up duration (average follow-up, 10.8 months) for patients implanted with this novel hearing device. However, insights from Cowan et al.’s investigation [17], which reported adverse events up to 24 months after surgery, indicated that the majority of such events manifested within the initial three months. This suggests that our study captures a significant portion of the post-operative adverse events timeline. 

### 4.5. Music Perception and Future Research Directions

Current trends in audiological rehabilitation emphasize high hearing functions, such as music listening. Both objective and subjective evaluations are utilized to measure the effectiveness of these rehabilitation methods. Notably, subjective evaluations have been shown to correlate with improvements in the quality of life, as indicated by Frosolini et al. [23] in their observational study on patient-reported outcome measures in cochlear implant patients.

Given the relevance of music rehabilitation for individuals with hearing loss and the use of advanced hearing devices like the Osia device, it is crucial to consider the potential benefits in this area. A relevant direction for future research is to examine the effect of the Osia device on high hearing functions. The study by Jiam et al. [24] provides a basis for this direction, highlighting the importance of music perception in users of bone-anchored hearing implants.

## 5. Conclusions

In conclusion, the Osia device emerges as a promising recommendation for individuals with conductive or mixed hearing loss, possibly also for those with SSD. Its safety and efficacy profile aligns with the broader category of active transcutaneous devices, demonstrating a reduced risk of wound infection compared to percutaneous alternatives. Our experience suggests that modifying the manufacturer-recommended incision can be beneficial in reducing incisional tension. Both audiological assessments and subjective evaluations revealed positive outcomes. Subsequent investigations should consider prolonged follow-up durations and larger sample sizes to comprehensively assess the utility and efficacy of this new transcutaneous BCHI.

## Figures and Tables

**Table 1:** Patient characteristics.

Patient	Sex	Age at Surgery (Y)	Type of Hearing Loss on the Implanted Side	Etiology	Implanted Ear	Type of Hearing Loss on the Non-Implanted Side	Follow-Up (Mo)	AC, Implanted Ear, (PTA_4_) (dB HL)	BC, Implanted Ear, (PTA_4_) (dB HL)
1	F	51	Mixed	Otitis chronica	sin.	Bilateral mixed	18	40, 20, 20, 45 (31)	20, 20, 30, 45 (29)
2	M	46	Conductive	Cholesteatoma bilat.	sin.	Bilateral conductive	16	30, 10, 20, 40 (25)	10, 15, 15, 25 (16)
3	F	52	Conductive	Cholesteatoma	sin.	Normal hearing	15	55, 25, 25, 35 (35)	25, 10, 20, 20 (19)
4	M	19	Conductive	Cholesteatoma	sin.	Normal hearing	15	35, 20, 25, 20 (25)	10, −5, 0, 10 (4)
5	F	42	Conductive	Cholesteatoma	dxt.	Normal hearing	15	30, 15, 10, 20 (19)	−10, 0, 0, 10 (0)
6	F	29	^a^SSD	Complication after tympanoplasty	dxt.	Normal hearing	12	Anacusis	Anacusis
7	M	17	Conductive	Microtia	dxt.	Normal hearing	12	35, 30, 15, 15 (24)	5, 15, 10, 5 (9)
8	F	63	Mixed	Otitis chronica	dxt.	Bilateral mixed	12	50, 45, 60, 75 (58)	30, 35, 55, 60 (45)
9	F	48	Conductive	Cholesteatoma	sin.	Normal hearing.	8	45, 25, 20, 40 (33)	20, 35, 20, 25 (25)
10	F	65	Mixed	Cholesteatoma	dxt.	Bilateral mixed	10	55, 50, 50, 60 (54)	25, 40, 55, 60 (45)
11	F	31	Conductive	Aural atresia	dxt.	Normal hearing	7	35, 20, 25, 25 (26)	15, 0, 10, 10 (9)
12	M	49	Conductive	Invasive invert papilloma	dxt.	Normal hearing	7	25, 20, 20, 25 (23)	5, 14, 10, 10 (10)
13	F	51	Conductive	Otosclerosis	sin.	Normal hearing	3	35, 20, 15, 25 (24)	0, 5, 0, −5 (0)
14	F	21	Conductive	Microtia	dxt.	Normal hearing	1 (^b^16)	30, 20, 20, 20 (22.5)	10, 10, 10, 5 (9)

AC = Air Conduction, BC = Bone Conduction, PTA_4_ = Pure Tone Average for the frequencies: 500 Hz, 1000 Hz, 2000 Hz and 4000 Hz, ^b^16 months follow up after first implantation, 1 month after second implantation. ^a^SSD: For the SSD patient AC non-implanted ear (PTA_4_): 10, 5, 10, 0 (6). BC non-implanted ear (PTA_4_): 5, 5, 5, 0 (1).

## Data Availability

The datasets generated and/or analyzed in the current study are available from the corresponding author upon reasonable request.
